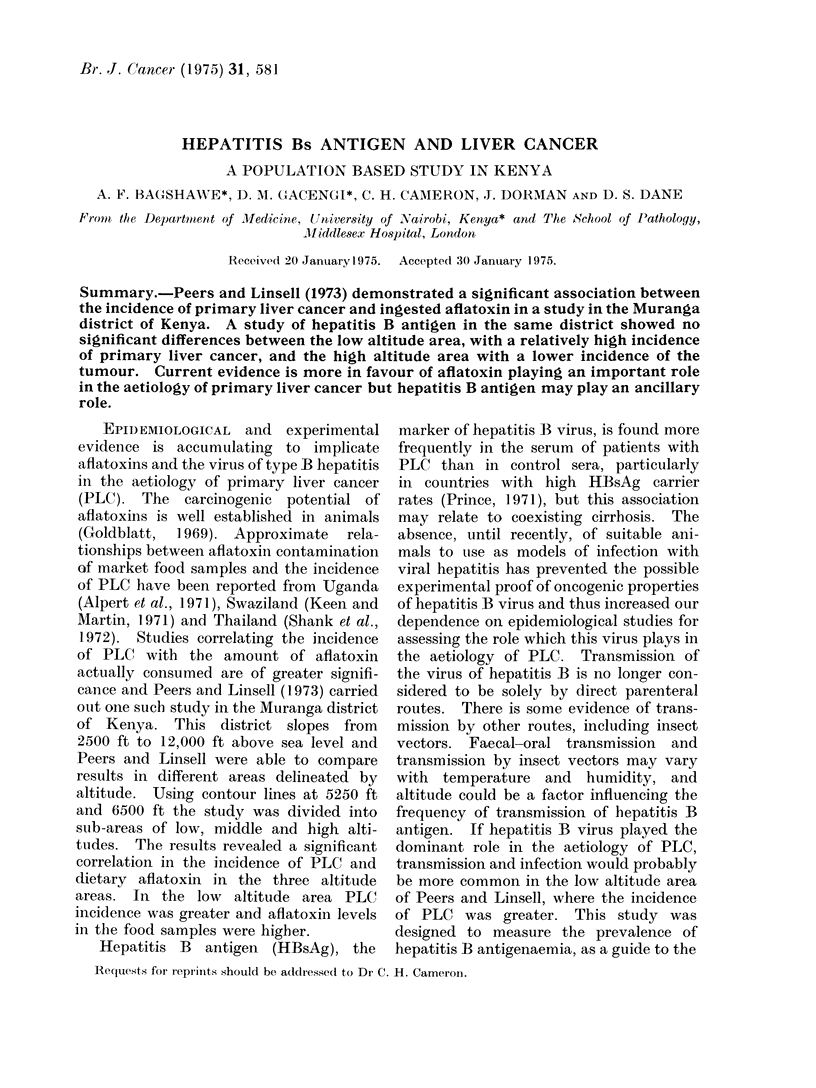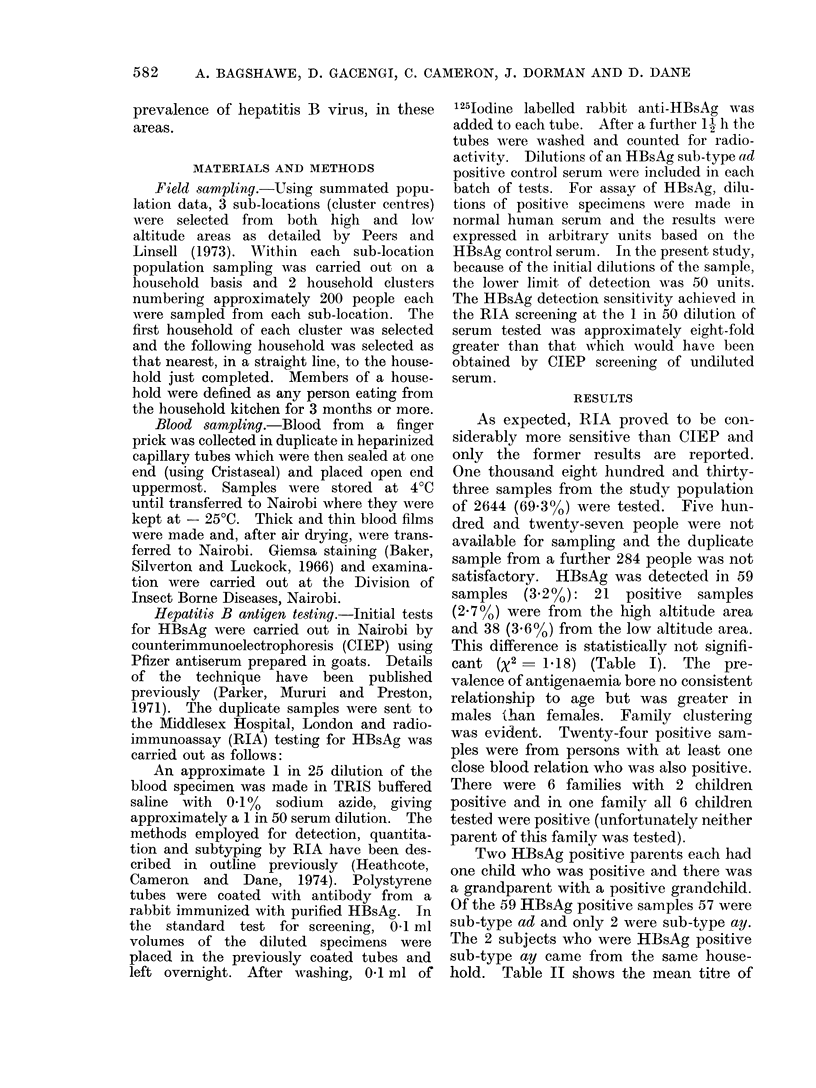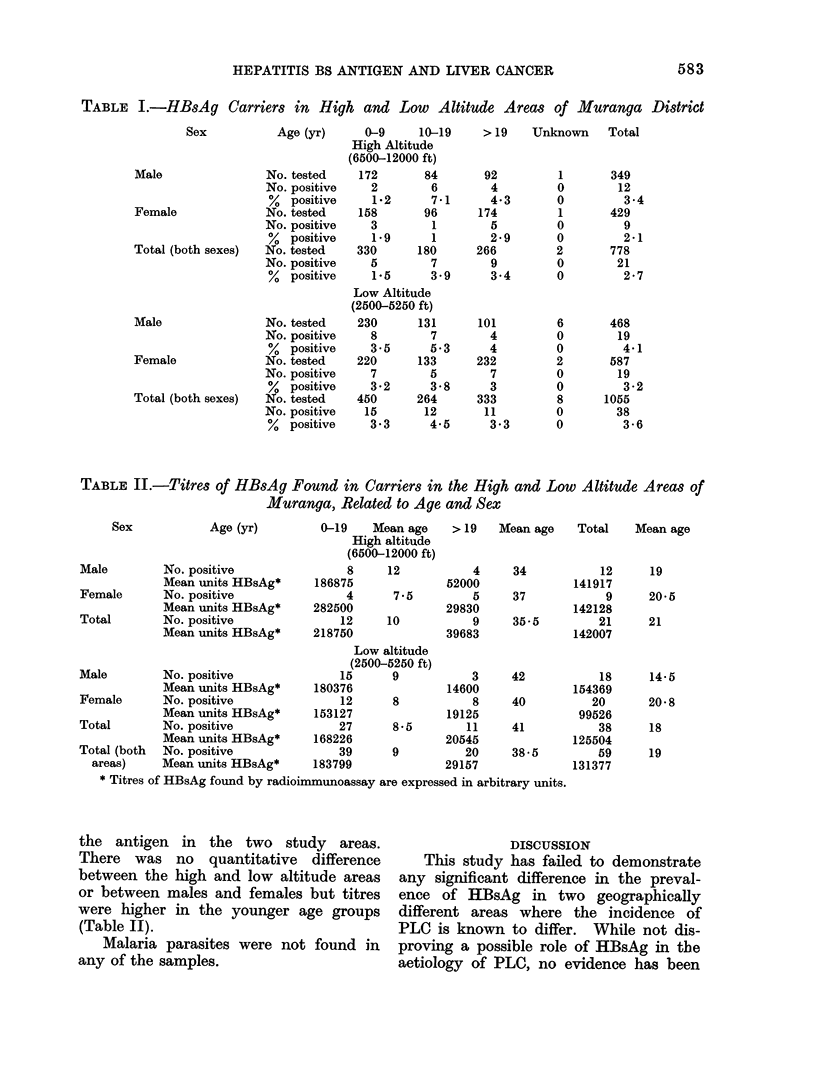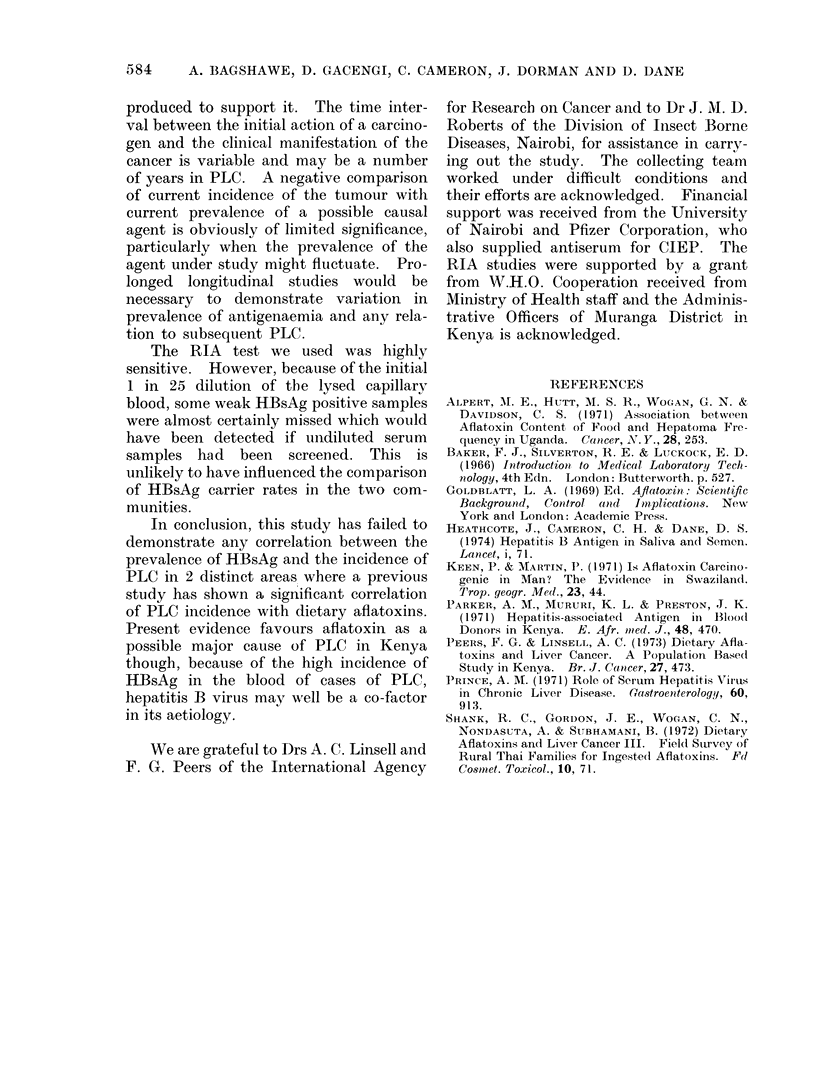# Hepatitis Bs antigen and liver cancer: A population based study in Kenya.

**DOI:** 10.1038/bjc.1975.99

**Published:** 1975-05

**Authors:** A. F. Bagshawe, D. M. Gacengi, C. H. Cameron, J. Dorman, D. S. Dane

## Abstract

Peers and Linsell (1973) demonstrated a significant association between the incidence of primary liver cancer and ingested aflatoxin in a study in the Muranga district of Kenya. A study of hepatitis B antigen in the same district showed no significant differences between the low altitude area, with a relatively high incidence of primary liver cancer, and the high altitude area with a lower incidence of the tumour. Current evidence is more in favour of aflatoxin playing an important role in the aetiology of primary liver cancer but hepatitis B antigen may play an ancillary role.


*Br. J. Cancer* (1975) **31**, 581

### HEPATITIS Bs ANTIGEN AND LIVER CANCER

### A POPULATION BASED STUDY IN KENYA

A. F. BAGSHAWE*, D. M. GACENGI*, C. H. CAMERON, J. DORMAN AND D. S. DANE

*From the Department of Medicine, University of Nairobi, Kenya* and The School of Pathology, Middlesex Hospital, London*

Received 20 January 1975. Accepted 30 January 1975.

**Summary.—Peers and Linsell (1973) demonstrated a significant association between the incidence of primary liver cancer and ingested aflatoxin in a study in the Muranga district of Kenya. A study of hepatitis B antigen in the same district showed no significant differences between the low altitude area, with a relatively high incidence of primary liver cancer, and the high altitude area with a lower incidence of the tumour. Current evidence is more in favour of aflatoxin playing an important role in the aetiology of primary liver cancer but hepatitis B antigen may play an ancillary role.**

EPIDEMIOLOGICAL and experimental evidence is accumulating to implicate aflatoxins and the virus of type B hepatitis in the aetiology of primary liver cancer (PLC). The carcinogenic potential of aflatoxins is well established in animals (Goldblatt, 1969). Approximate relationships between aflatoxin contamination of market food samples and the incidence of PLC have been reported from Uganda (Alpert *et al.*, 1971), Swaziland (Keen and Martin, 1971) and Thailand (Shank *et al.*, 1972). Studies correlating the incidence of PLC with the amount of aflatoxin actually consumed are of greater significance and Peers and Linsell (1973) carried out one such study in the Muranga district of Kenya. This district slopes from 2500 ft to 12,000 ft above sea level and Peers and Linsell were able to compare results in different areas delineated by altitude. Using contour lines at 5250 ft and 6500 ft the study was divided into sub-areas of low, middle and high altitudes. The results revealed a significant correlation in the incidence of PLC and dietary aflatoxin in the three altitude areas. In the low altitude area PLC incidence was greater and aflatoxin levels in the food samples were higher.

Hepatitis B antigen (HBsAg), the marker of hepatitis B virus, is found more frequently in the serum of patients with PLC than in control sera, particularly in countries with high HBsAg carrier rates (Prince, 1971), but this association may relate to coexisting cirrhosis. The absence, until recently, of suitable animals to use as models of infection with viral hepatitis has prevented the possible experimental proof of oncogenic properties of hepatitis B virus and thus increased our dependence on epidemiological studies for assessing the role which this virus plays in the aetiology of PLC. Transmission of the virus of hepatitis B is no longer considered to be solely by direct parenteral routes. There is some evidence of transmission by other routes, including insect vectors. Faecal–oral transmission and transmission by insect vectors may vary with temperature and humidity, and altitude could be a factor influencing the frequency of transmission of hepatitis B antigen. If hepatitis B virus played the dominant role in the aetiology of PLC, transmission and infection would probably be more common in the low altitude area of Peers and Linsell, where the incidence of PLC was greater. This study was designed to measure the prevalence of hepatitis B antigenaemia, as a guide to the

Requests for reprints should be addressed to Dr C. H. Cameron.

prevalence of hepatitis B virus, in these areas.

### MATERIALS AND METHODS

*Field sampling.*—Using summated population data, 3 sub-locations (cluster centres) were selected from both high and low altitude areas as detailed by Peers and Linsell (1973). Within each sub-location population sampling was carried out on a household basis and 2 household clusters numbering approximately 200 people each were sampled from each sub-location. The first household of each cluster was selected and the following household was selected as that nearest, in a straight line, to the household just completed. Members of a household were defined as any person eating from the household kitchen for 3 months or more.

*Blood sampling.*—Blood from a finger prick was collected in duplicate in heparinized capillary tubes which were then sealed at one end (using Cristaseal) and placed open end uppermost. Samples were stored at 4°C until transferred to Nairobi where they were kept at − 25°C. Thick and thin blood films were made and, after air drying, were transferred to Nairobi. Giemsa staining (Baker, Silverton and Luckock, 1966) and examination were carried out at the Division of Insect Borne Diseases, Nairobi.

*Hepatitis B antigen testing.*—Initial tests for HBsAg were carried out in Nairobi by counterimmunoelectrophoresis (CIEP) using Pfizer antiserum prepared in goats. Details of the technique have been published previously (Parker, Mururi and Preston, 1971). The duplicate samples were sent to the Middlesex Hospital, London and radioimmunoassay (RIA) testing for HBsAg was carried out as follows:

An approximate 1 in 25 dilution of the blood specimen was made in TRIS buffered saline with 0·1% sodium azide, giving approximately a 1 in 50 serum dilution. The methods employed for detection, quantitation and subtyping by RIA have been described in outline previously (Heathcote, Cameron and Dane, 1974). Polystyrene tubes were coated with antibody from a rabbit immunized with purified HBsAg. In the standard test for screening, 0·1 ml volumes of the diluted specimens were placed in the previously coated tubes and left overnight. After washing, 0·1 ml of $^{125}$Iodine labelled rabbit anti-HBsAg was added to each tube. After a further $1\frac{1}{2}$ h the tubes were washed and counted for radioactivity. Dilutions of an HBsAg sub-type *ad* positive control serum were included in each batch of tests. For assay of HBsAg, dilutions of positive specimens were made in normal human serum and the results were expressed in arbitrary units based on the HBsAg control serum. In the present study, because of the initial dilutions of the sample, the lower limit of detection was 50 units. The HBsAg detection sensitivity achieved in the RIA screening at the 1 in 50 dilution of serum tested was approximately eight-fold greater than that which would have been obtained by CIEP screening of undiluted serum.

### RESULTS

As expected, RIA proved to be considerably more sensitive than CIEP and only the former results are reported. One thousand eight hundred and thirty-three samples from the study population of 2644 (69·3%) were tested. Five hundred and twenty-seven people were not available for sampling and the duplicate sample from a further 284 people was not satisfactory. HBsAg was detected in 59 samples (3·2%): 21 positive samples (2·7%) were from the high altitude area and 38 (3·6%) from the low altitude area. This difference is statistically not significant ($\chi^2 = 1{\cdot}18$) (Table I). The prevalence of antigenaemia bore no consistent relationship to age but was greater in males than females. Family clustering was evident. Twenty-four positive samples were from persons with at least one close blood relation who was also positive. There were 6 families with 2 children positive and in one family all 6 children tested were positive (unfortunately neither parent of this family was tested).

Two HBsAg positive parents each had one child who was positive and there was a grandparent with a positive grandchild. Of the 59 HBsAg positive samples 57 were sub-type *ad* and only 2 were sub-type *ay*. The 2 subjects who were HBsAg positive sub-type *ay* came from the same household. Table II shows the mean titre of

TABLE I.—*HBsAg Carriers in High and Low Altitude Areas of Muranga District*

| Sex | Age (yr) | 0–9 | 10–19 | > 19 | Unknown | Total |
|---|---|---|---|---|---|---|
| | | High Altitude (6500–12000 ft) | | | | |
| Male | No. tested | 172 | 84 | 92 | 1 | 349 |
| | No. positive | 2 | 6 | 4 | 0 | 12 |
| | % positive | 1·2 | 7·1 | 4·3 | 0 | 3·4 |
| Female | No. tested | 158 | 96 | 174 | 1 | 429 |
| | No. positive | 3 | 1 | 5 | 0 | 9 |
| | % positive | 1·9 | 1 | 2·9 | 0 | 2·1 |
| Total (both sexes) | No. tested | 330 | 180 | 266 | 2 | 778 |
| | No. positive | 5 | 7 | 9 | 0 | 21 |
| | % positive | 1·5 | 3·9 | 3·4 | 0 | 2·7 |
| | | Low Altitude (2500–5250 ft) | | | | |
| Male | No. tested | 230 | 131 | 101 | 6 | 468 |
| | No. positive | 8 | 7 | 4 | 0 | 19 |
| | % positive | 3·5 | 5·3 | 4 | 0 | 4·1 |
| Female | No. tested | 220 | 133 | 232 | 2 | 587 |
| | No. positive | 7 | 5 | 7 | 0 | 19 |
| | % positive | 3·2 | 3·8 | 3 | 0 | 3·2 |
| Total (both sexes) | No. tested | 450 | 264 | 333 | 8 | 1055 |
| | No. positive | 15 | 12 | 11 | 0 | 38 |
| | % positive | 3·3 | 4·5 | 3·3 | 0 | 3·6 |

TABLE II.—*Titres of HBsAg Found in Carriers in the High and Low Altitude Areas of Muranga, Related to Age and Sex*

| Sex | Age (yr) | 0–19 | Mean age | > 19 | Mean age | Total | Mean age |
|---|---|---|---|---|---|---|---|
| | | High altitude (6500–12000 ft) | | | | | |
| Male | No. positive | 8 | 12 | 4 | 34 | 12 | 19 |
| | Mean units HBsAg* | 186875 | | 52000 | | 141917 | |
| Female | No. positive | 4 | 7·5 | 5 | 37 | 9 | 20·5 |
| | Mean units HBsAg* | 282500 | | 29830 | | 142128 | |
| Total | No. positive | 12 | 10 | 9 | 35·5 | 21 | 21 |
| | Mean units HBsAg* | 218750 | | 39683 | | 142007 | |
| | | Low altitude (2500–5250 ft) | | | | | |
| Male | No. positive | 15 | 9 | 3 | 42 | 18 | 14·5 |
| | Mean units HBsAg* | 180376 | | 14600 | | 154369 | |
| Female | No. positive | 12 | 8 | 8 | 40 | 20 | 20·8 |
| | Mean units HBsAg* | 153127 | | 19125 | | 99526 | |
| Total | No. positive | 27 | 8·5 | 11 | 41 | 38 | 18 |
| | Mean units HBsAg* | 168226 | | 20545 | | 125504 | |
| Total (both areas) | No. positive | 39 | 9 | 20 | 38·5 | 59 | 19 |
| | Mean units HBsAg* | 183799 | | 29157 | | 131377 | |

* Titres of HBsAg found by radioimmunoassay are expressed in arbitrary units.

the antigen in the two study areas. There was no quantitative difference between the high and low altitude areas or between males and females but titres were higher in the younger age groups (Table II).

Malaria parasites were not found in any of the samples.

### DISCUSSION

This study has failed to demonstrate any significant difference in the prevalence of HBsAg in two geographically different areas where the incidence of PLC is known to differ. While not disproving a possible role of HBsAg in the aetiology of PLC, no evidence has been produced to support it. The time interval between the initial action of a carcinogen and the clinical manifestation of the cancer is variable and may be a number of years in PLC. A negative comparison of current incidence of the tumour with current prevalence of a possible causal agent is obviously of limited significance, particularly when the prevalence of the agent under study might fluctuate. Prolonged longitudinal studies would be necessary to demonstrate variation in prevalence of antigenaemia and any relation to subsequent PLC.

The RIA test we used was highly sensitive. However, because of the initial 1 in 25 dilution of the lysed capillary blood, some weak HBsAg positive samples were almost certainly missed which would have been detected if undiluted serum samples had been screened. This is unlikely to have influenced the comparison of HBsAg carrier rates in the two communities.

In conclusion, this study has failed to demonstrate any correlation between the prevalence of HBsAg and the incidence of PLC in 2 distinct areas where a previous study has shown a significant correlation of PLC incidence with dietary aflatoxins. Present evidence favours aflatoxin as a possible major cause of PLC in Kenya though, because of the high incidence of HBsAg in the blood of cases of PLC, hepatitis B virus may well be a co-factor in its aetiology.

We are grateful to Drs A. C. Linsell and F. G. Peers of the International Agency for Research on Cancer and to Dr J. M. D. Roberts of the Division of Insect Borne Diseases, Nairobi, for assistance in carrying out the study. The collecting team worked under difficult conditions and their efforts are acknowledged. Financial support was received from the University of Nairobi and Pfizer Corporation, who also supplied antiserum for CIEP. The RIA studies were supported by a grant from W.H.O. Cooperation received from Ministry of Health staff and the Administrative Officers of Muranga District in Kenya is acknowledged.

### REFERENCES

Alpert, M. E., Hutt, M. S. R., Wogan, G. N. & Davidson, C. S. (1971) Association between Aflatoxin Content of Food and Hepatoma Frequency in Uganda. *Cancer, N.Y.*, **28**, 253.

Baker, F. J., Silverton, R. E. & Luckock, E. D. (1966) *Introduction to Medical Laboratory Technology*, 4th Edn. London: Butterworth. p. 527.

Goldblatt, L. A. (1969) Ed. *Aflatoxin: Scientific Background, Control and Implications.* New York and London: Academic Press.

Heathcote, J., Cameron, C. H. & Dane, D. S. (1974) Hepatitis B Antigen in Saliva and Semen. *Lancet*, i, 71.

Keen, P. & Martin, P. (1971) Is Aflatoxin Carcinogenic in Man? The Evidence in Swaziland. *Trop. geogr. Med.*, **23**, 44.

Parker, A. M., Mururi, K. L. & Preston, J. K. (1971) Hepatitis-associated Antigen in Blood Donors in Kenya. *E. Afr. med. J.*, **48**, 470.

Peers, F. G. & Linsell, A. C. (1973) Dietary Aflatoxins and Liver Cancer. A Population Based Study in Kenya. *Br. J. Cancer*, **27**, 473.

Prince, A. M. (1971) Role of Serum Hepatitis Virus in Chronic Liver Disease. *Gastroenterology*, **60**, 913.

Shank, R. C., Gordon, J. E., Wogan, C. N., Nondasuta, A. & Subhamani, B. (1972) Dietary Aflatoxins and Liver Cancer III. Field Survey of Rural Thai Families for Ingested Aflatoxins. *Fd Cosmet. Toxicol.*, **10**, 71.